
JOURNAL INFORMATION
==============================
NLM Title Abbreviation: Sci China Life Sci
Journal ID: Sci China Life Sci
NLM Journal ID: 101529880

Science China. Life Sciences

ISSN: 1674-7305
EISSN: 1869-1889

ARTICLE INFORMATION
==============================
PMCID: PMC7088622
PMID: 24526494
DOI: 10.1007/s11427-014-4625-2
Article ID: 4625
Article version: 1
Subjects: Insight

Implementing immunization program efficiently, and establishing national database of adverse reactions

Gao George Fu gaofu@chinacdc.cn

grid.198530.6 0000000088032373 Chinese Center for Disease Control and Prevention, Beijing, 102206 China
Electronic publication date: 2014 Feb 13
Print publication date: 2014
Volume: 57
Issue: 5
First page: 553
Last page: 554
Received 2014 Jan 12; Accepted 2014 Jan 27
Copyright: © The Author(s) 2014
License: Open AccessThis article is distributed under the terms of the Creative Commons Attribution 4.0 International License (https://creativecommons.org/licenses/by/4.0), which permits use, duplication, adaptation, distribution, and reproduction in any medium or format, as long as you give appropriate credit to the original author(s) and the source, provide a link to the Creative Commons license, and indicate if changes were made.
License URL: https://creativecommons.org/licenses/by/4.0/

Keywords: Measle, Pertussis, Smallpox, Measle Vaccine, National Immunization Program
issue-copyright-statement: © Science in China Press and Springer-Verlag GmbH 2014

==============================

GAO George Fu

In the end of 2013, an official response was issued to address reports by the local media that several children had died following vaccination with the hepatitis B vaccine. Such reports had aroused considerable suspicion from the public regarding the safety and quality of vaccines. The State Food and Drug Administration (SFDA) decided to temporarily suspend the use of the hepatitis B vaccine manufactured by Shenzhen Kangtai Biological Products Co., Ltd. The withdrawal of this vaccine only heightened public concern about potential "unqualified vaccines" manufactured by Kangtai. When issuing the above decision, the SFDA announced that it would take approximately 20 days to complete testing and to issue the final results. The fallout from this event could be damaging for other immunization programs, as public confidence in the effectiveness and need for vaccination strategies wanes. A joint statement issued by the SFDA and National Health And Family Planning Commission on January 3, 2014 stated no quality issues were found for the hepatitis B vaccine manufactured by Kangtai. In addition, of the 17 deaths linked to vaccination with the hepatitis B vaccine, nine deaths were confirmed to have no association with the vaccine, whilst preliminary diagnosis of the remaining eight cases suggests their death is also unrelated to the vaccine. The scope of the impact resulting from public suspicion of the hepatitis B vaccine and immunization programs on the whole, cannot be underestimated. This event highlights the need and urgency for China to improve their efforts in risk communication.

In short, we should adopt a calm, non-emotive and logical approach in our assessment of this event. We should not implement changes to the national vaccination program based on a single "media-misleading" event. A review of vaccine development yielded records of human variolation for smallpox prevention in a number of Chinese history books, but the smallpox vaccination invented by a British doctor, Edward Jenner, has been universally regarded as the origin of vaccination and current immunology. Thanks to Jenner's invention, smallpox has been eradicated, and immunology (a relatively new scientific branch) was developed. Immunology has played a significant role in controlling pathogenic microorganisms and tumors. Vaccines developed against various pathogenic microorganisms have invariably saved numerous lives, in both developed and developing countries. The national immunization program implemented in China includes 14 vaccines against 15 infectious diseases, and to date it has played an integral role in disease prevention and control in China. Therefore, the effectiveness of vaccines and the necessity for immunization in disease control and prevention should not be overlooked because of this one event.

In general, increasing suspicion and lack of confidence in vaccine immunizations are occurring globally. Resistance to vaccination with PDT (pertussis, diphtheria, and tetanus) and/or measles vaccines in the Great Britain resulted in an increase in reportable pertussis and measles cases. The topic for World Immunization Week (the last week in April of every year) is as follows: "understand the situation and conduct vaccination". The purpose of setting up World

Immunization Week is to highlight the roles played by vaccines in preventing and controlling infectious diseases.

Hepatitis B vaccine events do provide warnings and several suggestions for future work, which are presented as follows:

(i) The media and management should address how they communicate serious health risks to the public. In particular, all information communicated to the public should be based on facts to avoid inciting panic. Recent discussions on genetically modified organisms highlight such a lack of rational communication. We need to better educate the public on the historical role of vaccines, and the critical roles of vaccines in disease control and prevention. In most cases, vaccination confers a lifetime protection against diseases.

(ii) We should establish a national vaccine adverse reaction database. Municipalities and provinces with sound information infrastructure, such as Shanghai and Zhejiang, should take the lead in the establishment of such a database; with the intention of extending this throughout the whole country.

(iii) We should establish an honest and reliable confirmation and compensation mechanism for adverse reactions. This would hopefully renew public confidence in research related to vaccine production, circulation, management, inoculation and follow-up services.

(iv) In general, there is a lack of public knowledge (public understanding of science) of vaccines and immunization, whilst scientific dissemination of vaccines by researchers and producers is of reduced importance. It is hoped that by making available more scientific-based literature on vaccines to the wider community, the lay-person would develop a greater understanding of the importance of vaccines in disease control and prevention, and consequently the need for further research in this area.

(v) A nationwide epidemiological survey of hepatitis B infection should be undertaken, as the last study was conducted in 1992. We hope that the relevant departments can take prompt action to thoroughly investigate the current domestic epidemics and infections, strengthen the relevant basic research and adjust immunization strategies based on sound scientific data. We should consider whether the current immunization strategies (programs) are practical for a hepatitis B negative family whose child will be vaccinated within 24 h after birth, and whether the time of this first inoculation could be changed.

**Biographical Sketch**

**Open Access** This article is distributed under the terms of the Creative Commons Attribution License which permits any use, distribution, and reproduction in any medium, provided the original author(s) and source are credited.

This article is published with open access at link.springer.com

Biographical Sketch

Gao George Fu obtained his Ph.D. degree from Oxford University, UK and did his postdoc work in both Oxford University and Harvard University (with a brief stay in Calgary University). He is currently deputy director-general of Chinese Center for Disease Control and Prevention and vice-president of Beijing Institutes of Life Science, Chinese Academy of Sciences. He is also the director of CAS Key Laboratory of Pathogenic Microbiology and Immunology. His research interests include enveloped viruses and molecular immunology. His group research is focusing on the enveloped virus entry and release, especially influenza virus interspecies transmission (host jump), structure-based drug-design and structural immunology. He has published more than 260 refereed papers, 10 books or book chapters and has applied and obtained more than 25 UK, US and Chinese patents. His recent work on HA/receptor binding and structural basis of both H7N9 and H5N1 influenza viruses provided novel insights into the molecular mechanism of avian-flu “host jump” and the work on MERS-CoV entry delineated the molecular mechanism of receptor-viral protein interaction (Science, 2013a, 2013b; The Lancet, 2013; Nature Communications, 2014; Nature, 2013). He has obtained numerous awards, including TWAS Medical Prize (2012). Professor Gao was elected a CAS member (academician) in 20

